# Organizational practices promoting employees’ pro-environmental behaviors in a Visegrad Group country: How much does company ownership matter?

**DOI:** 10.1371/journal.pone.0261547

**Published:** 2022-02-03

**Authors:** Katarzyna Piwowar-Sulej, Izabela Kołodziej

**Affiliations:** Department of Labor, Capital and Innovation, Wroclaw University of Economics and Business, Wroclaw, Poland; University of Hradec Kralove: Univerzita Hradec Kralove, CZECH REPUBLIC

## Abstract

Although company ownership (local vs. foreign) has been used as a contingency variable that differentiates companies’ environmental activities and performance, the current understanding of these differences is fragmented. None of the previous studies examined the relationships between company ownership and a complex set of organizational practices which promote employees’ pro-environmental behaviors. The article fills the gap in previous research by analyzing the extent to which a total of 37 organizational practices are used. These practices are divided into technical (20 are related to environmental management) and “soft” (17 are related to HRM policies). For this study, both literature studies and a survey covering 199 companies located in Poland were used. In the theoretical part, the paper presents various typologies of factors that enhance employees’ green behavior, with the focus on organizational practices. There is also a review of studies on environmental sustainability which used company ownership as a variable. The results of the empirical research show that–although in foreign companies environmental issues are increasingly perceived as important or very important–many of the practices which are treated as crucial for developing employees’ eco-friendly behaviors are rarely used in both local and foreign companies. Moreover, company ownership was important in the context of using 88% of the “soft” practices and 50% of the technical practices discussed in the article. The article provides implications for policy and practice, as well as directions for further research.

## Introduction

The idea of sustainability was created in the 1960s as a response to such problems as environmental pollution, excessive consumption of natural resources, and destruction of the Earth’s surface (especially forests), as well as a high unemployment rate and malnutrition. In 1997, Elkington [1] identified three fundamental principles on which sustainable development is based: environmental integrity (planets), social justice (people), and economic wellbeing (prosperity).

The 2007 report by the Intergovernmental Panel on Climate Change [2] concluded that the drivers of climate change are currently a very serious problem in terms of global warming. It also indicates that the total global greenhouse gas emissions from industry are three times higher than those emitted by households. According to Khan et al. [3], the activities of companies are considered one of the major factors contributing to this threat; however, business is beginning to be perceived as a solution to, rather than a cause of, environmental degradation [4, 5].

Implementing the idea of sustainable development in companies would not be possible without appropriate employee behavior. Therefore, more and more attention is being paid to the issue of developing pro-ecological behavior (PEB; also known as green, pro-environmental, environmentally-friendly, or eco-friendly behavior) in the workplace [6, 7]. Such behaviors are consciously used to minimize the negative impact of one’s actions on the environment [8].

Organizational practices promoting PEB are mostly in the area of HRM. They will be called “soft.” As pointed out by Paillé, Raineri, and Boiral [9], activities falling within environmental management are also important. They are more of a technical nature, as noted by Wagner [10], and therefore will be called “technical”.

Companies differ in terms of the activities they undertake to protect the natural environment. The differences are justified with the use of stakeholder theory [11, 12]. Freeman–who implemented a stakeholder approach to strategic management–defined stakeholders as “any group or individual who can affect or is affected by the achievement of the organization’s objectives” [13] (p. 46). The literature about environmentalism presents a typology of stakeholders which distinguishes such entities as government (regulatory bodies with high power and high legitimacy), media (with high urgency and low legitimacy), clients (with high power and salience), company owners and managing boards (with high power), suppliers (with low power, low legitimacy, and low urgency), and employees (with moderate power, legitimacy, and urgency) [14]. It was proven that stakeholder-centric governance positively impacts organizational performance [15]. Since organizational environmental responsibility is actively stimulated, by country-level legal regulations and trade and industry associations, among other things [16], there are differences in green practices between companies of different size, and operating in different countries and industries.

One of the variables used in research on environmental sustainability is company ownership (local vs. foreign). Foreign ownership means that a business in a country is owned/controlled by a company/companies whose headquarters are not in that country or by individuals who are not citizens of that country [17]. Previous studies examined the relationships between this variable and such variables as environmental commitment and waste management [18], the implementation of ISO 14001 standards [19], and the use of environmental training [20]. As Balasubramanian et al. (2021) stated, the current understanding of relationships between company ownership and environmental practices is “fragmented and scattered across the literature.” None of the previous studies covering company size examined a broad range of organizational practices that promote PEB. This constitutes an important premise for undertaking research that addresses this problem.

The purpose of this article is to answer two research questions:

1. What are the differences between local and foreign companies regarding which technical practices they apply in order to develop employees’ PEB?
2. What are the differences between local and foreign companies regarding which “soft” practices they apply in order to develop employees’ PEB?

As Zibarras and Coan [21] stated, “research uncovering how businesses structure their policies and initiatives to enhance the opportunities for environmental sustainability is now critical” (p. 2). This should be applied to various countries and regions. Research on environmental sustainability which used company ownership as a variable has never directly addressed Poland, one of the Visegrad Group countries [12]. Among the scientific publications indexed in the Scopus and Web of Sciences databases, only one is devoted to practices developing PEB in young Polish companies [22].

Poland is the geographical area of scientific interest covered in this article. Poland’s membership in the EU imposes numerous obligations regarding standards for environmental protection, though according to Eurostat’s (2020) [23] data, Poland ranks below the average for all EU countries in various environmental indicators. Moreover, research shows that only one third of Poles act to protect their health in terms of air quality in their homes. When buying beverages in glass packages, 70% of citizens do not pay attention to whether the bottle is suitable for return.

This article contributes to the literature on the subject by 1) collating and analyzing different factors used as stimulators of employees’ PEB with a focus on organizational practices, 2) providing quantitative research results that include a variable for the origin of capital and examine a set of technical and “soft” organizational practices which promote employees’ PEB, and 3) formulating further research directions.

This study is organized into five sections. After the introduction, the second section presents the literature background. It discusses factors that influence employees’ PEB as well as previous research which used a variable for the company’s ownership. The third section describes the research methodology. In the fourth section, the authors present the results of their empirical research, followed by a discussion and practical implications of them. The article ends with the main conclusions, limitations of the study, and directions for further research.

## Literature background

### Factors that influence employees’ PEB, focusing on organizational practices

The literature on environmental sustainability presents various approaches to factors that influence the environmentally-friendly behavior of employees (Table 1).

**Table 1:** Factors that influence employees’ PEB.

Authors	Factors
Govindarajulu and Daily [24]	• commitment of management
	• rewards
	• empowerment
	• feedback and review
Wagner [10]	• technical activities (e.g., green design of products)
	• organizational activities (e.g., environmental policy or clearly defined responsibilities)
Xie et al. [25]	• green office building
Muster and Schrader [26]	• green work–life balance
Zibarras et al. [27]	• environmental initiatives (e.g., environmental policy or green IT)
Renwick et al. [28]	• green hiring
	• green training
	• assessing employees by considering PEB
	• rewarding such behavior
Tariq et al. [29]	• employee empowerment
De Prins [30]	• horizontal practices: employee participation and empowerment, care about health and wellbeing
	• vertical practices: recruitment, selection, training, development, compensation, change, leadership, and culture
Kim et al. [31]	• autonomous motivation
	• controlled motivation (extrinsic)
Haddock-Millar et al. [32], Afsar et al. [33]	• managers’ behavior
Křepelková et al. [34]	• experiences of contact with nature in childhood
Wiernik, Dilchert, and Ones [35]	• individual factor–employees’ age
	• contextual factors
Ruepert, Keizer, and Steg [36]	• contextual factors
	• employee values
Khan et al. [3]	• the atmosphere in the workplace
	• organizational justice
	• green HRM
	• motivating
	• green training
	• feedback from managers
	• ecological production
Zientara et al. [37]	• values in society
	• individual factors
	• education
Lu et al. [38]	• pro-environmental values
	• appropriate company strategy
Paillé, Raineri, and Boiral [9]	• contextual factors (environmental management practices)
	• psychological factors (employees’ personal environmental beliefs, self-efficacy, and emotional commitment)
Yunguo [39], Gürlek and Tuna [40], Wang [41], Piwowar-Sulej [42], Shafaei et al. [43], Magill et al. [44]	• green organizational culture (or climate)
Al-Ghazali and Afsar [45]	• green recruitment and selection
	• green training and development
	• green performance management and appraisal
	• green reward and compensation
	• green empowerment and participation
	• green creativity (introducing brainstorming to create pro-environmental solutions)
	• green behavioral intention and values (personal characteristics)
Yuriev and Sierra-Barón [46]	• behavioral beliefs
	• normative beliefs
	• controlling beliefs

Various authors have highlighted the importance of different factors in developing PEB. Generally, these factors can be divided into three groups. The first group covers factors related to experiences in ones’ private life. The second group includes the activities performed by an organization. Finally, the third group of factors is related to the individual characteristics of an employee (e.g., demographic or psychological [47]).

It is worth emphasizing here that the issue of factors influencing employees’ behaviors is especially examined by researchers in the area of human resources management (HRM). General practices which promote the sustainable use of business resources and prevent harm arising from environmental concerns are part of the definition of *green HRM* [48]. However, some authors have adopted a narrower definition of green HRM, in which the practices are closely related to the functional areas of HRM, such as recruitment and selection, training, compensation, and development [49, 50].

Green recruitment and selection are designed to provide a company with highly qualified employees who show a high degree of environmental awareness, whereas the subsequent practices refer to developing appropriate behavior among those already employed.

Stimulating PEB among existing personnel takes place within green HR performance management, for example. Pinzone et al. [6] proved the effectiveness of HR appraisal in employees’ collective organizational citizenship PEB. Gholami et al. [51] also emphasized the importance of HR performance appraisal.

The importance of a compensation system in promoting PEB was highlighted by Florida and Davidson [52]. Gholami et al. [51] demonstrated that a pay and reward system is ranked second in green HRM systems’ hierarchy. Ramus [53] and Handgraaf et al. [54] found that recognition-based rewards (e.g., plaques or letters of praise) had a better impact on employees’ pro-environmental commitment than other types of rewards.

Zientara et al. [37] linked employee education to individual factors, such as beliefs or awareness. They indicated that people who are aware of environmental threats may be more eager to segregate and recycle waste, purchase ecological products, or commute to work by public transport. This is related to employee training. Daily and Huang [55] found that training and rewards are the key factors responsible for effective environmental management. Fernández et al. [56], Renwick et al. [28], and Zibarras and Coan [21] also emphasized the importance of these two factors by highlighting their significant role in building pro-environmental awareness and a pro-environmental culture.

When discussing various initiatives undertaken to educate employees, it is worth noting that Jackson and Seo [57] provided examples of companies that build employee gardens, thus stimulating employee gardening activities and, as a result, teaching employees what environmental sustainability means. Likewise, Xie et al. [25] emphasized the importance of green office buildings in promoting PEB. In turn, Oppong-Tawiah et al. [7] promote gamification.

To continue the discussion on organizational practices which promote PEB, it should be stated that some authors go beyond the classic HR function and emphasize the importance of green participation and empowerment [24, 29, 58]. The specific practices related to green participation include handling internal communication regarding “green” initiatives, establishing a forum for exchanging opinions and environmental knowledge, creating “green teams,” engaging employees in developing the organization’s environmental strategy, and defining both goals for the team and methods for measuring the degree to which they are achieved [59]. Participation–as with training–is closely related to releasing the creative potential of employees. Managers play a significant role here. They should provide regular feedback to employees or teams about their role in achieving the environmental goals [30, 60], encourage their subordinates to report problems and solutions, and delegate their authority.

Organizational practices do not only take the form of activities in the area of HRM. As pointed out by Wagner [10] and Paillé, Raineri, and Boiral [9], activities falling under environmental management are important. They are more of a technical nature. An appropriate environmental strategy and policy is the key to achieving PEB among employees [38]. Other–more technical practices–include the development of green technologies and products, the improvement of energy efficiency, and the recycling of waste. Ruepert, Keizer, and Steg [36] referred to such practices as contextual factors. In their model, they showed that people are more willing to take up pro-ecological activities when the appropriate context of the situation in which they find themselves focuses them on the environmental aspects.

Finally, it is the organizational culture that combines all of the above-mentioned practices. Green organizational culture is an element of organizational culture (e.g., values and norms) that reflects the organization’s environmental concerns. The level of green organizational culture is influenced by all of the above-mentioned organizational practices and individual characteristics presented by employees [42].

### Firm ownership as a variable used in previous research on environmental sustainability

Literature studies were conducted through a search of articles in the Scopus database using such queries as (“environmental” or “green”) and "practices" and "firm ownership". The titles, abstracts, and keywords were searched. The final sample covered only 24 documents. After scanning the resulting documents, only those which focused on local vs. foreign ownership underwent further analysis (e.g., articles on family-owned and government-owned businesses were excluded from further analysis). The authors also analyzed the articles presented by Balasubramanian et al. [12] in their literature review on company characteristics and environmental sustainability.

As S1 Table shows, there is much more research on differences between local and foreign companies in the use of technical environmental practices than on green HRM practices.

Previous research was conducted in various countries. For example, the study by Eltayeb and Zailani [61] covered manufacturing companies which had implemented ISO 14001 in Malaysia. The results showed that the green supply chain initiatives vary depending on the ownership type. The lowest level in this respect was recorded in Malaysian local companies. In terms of foreign companies, the study found that Japanese and European companies featured a higher level of green supply chain initiatives than US companies. In turn, King and Shaver [62] observed that the companies owned by foreign entities located in the USA generate more waste (i.e., American local companies are more green).

Faith et al. [18] also examined green purchasing, but in the construction industry in Nigeria. Foreign construction companies, much more often than domestic ones, realized the importance of using environmentally friendly materials and conducting environmental audits. In turn, Albornoz et al. [63] conducted their research in Argentina. They found that the foreign-owned companies were implementing more practices related to environmental management than the local ones. A study by Christmann and Taylor [19] in China showed that the international property positively influences the compliance with environmental regulations as well as the likelihood of ISO 14000 implementation. Similar studies were carried out by Zhu, Cordeiro, and Sarkis [64], focusing on manufacturing companies in China. The authors confirmed the hypothesis that both foreign companies and joint ventures adopted ISO 14001 and total quality environmental management more often, whereas eco-auditing practices were less frequent.

The difference between various forms of company ownership introducing environmental management systems was not confirmed by Andonova [65] and Garcia et al. [66], who based their studies on secondary data from the Harvard Institute for International Development. In 1998, this institute researched the most polluting industrial sectors in Central and Eastern European post-socialist countries. They found that international capital did not result in a faster introduction of clean technologies. This observation is contradictory to the widespread expectation in transition countries that international capital will be a major source of modernization and technological innovation.

## Research methodology

This research project was conducted from January to October 2020. It was financed by the Ministry of Science and Higher Education in Poland under the program "Regional Initiative of Excellence" 2019–2022 project number 015/RID/2018/19 total funding amount 10 721 040,00 PLN. The purpose of this research was to answer the research questions presented in the Introduction. Moreover, the following two research hypotheses were formulated:

H1: There are statistically significant differences between local companies and foreign-owned ones in the extent to which most technical practices for developing employees’ PEB are applied.
H2: There are statistically significant differences between local companies and foreign-owned ones in the extent to which most of the “soft” practices for developing employees’ PEB are applied.

The authors used the survey method while conducting the empirical research. The survey instrument (a questionnaire) was based on the questions and scales by Zibarras et al. [27], which was originally addressed to respondents in the UK. It means that respondents marked whether technological activities are applied in their companies by choosing between “yes,” “no,” and “I don’t know.” In turn, concerning green HRM, the respondents marked the frequencies of individual practices by choosing between “never,” “rarely,” and “often.” The first question in the survey (introducing the topic) was as follows: “How important are environmental issues for your organization?” The respondents could choose their answer from the options “unimportant,” “moderately important,” or “very important”.

The data were collected between March and May 2020. The electronic questionnaire was sent to university graduates working in companies from various industries, with subsequent snowball sampling. This method is based on non-random sample selection–more precisely, on the study participants recruiting other participants. Researchers use this method when it is difficult to locate participants for the survey [67]. Although this method was originally applied in typically qualitative research, nowadays it is also accepted in quantitative research, primarily in situations where it is impossible to construct a random research sample [68]. In this study, the respondents recruited other respondents with a similar educational level and job title (line employee or middle-level manager) for the study. The researchers wanted the respondents to understand the specific nature of the problems covered by the research.

A total of 199 valid questionnaires were collected. All the respondents voluntarily participated in this study. The research was approved by the Rector Commission on Research Ethics functioning at the Wrocław University of Economics and Business (written consent no. 3A/2021) and the raw data is archived in an open repository (doi: 10.18150/SLZOHA).

The authors checked the names of companies in order to exclude multiple surveys from one organization, which would have reduced the research quality. In this way, diversity among the sample group was achieved, which is a necessary condition that ensures the validity of the research [68]. Table 2 presents the characteristics of the research sample.

**Table 2:** Characteristics of the research sample.

Criterion	Item	Number of representatives in the research sample	Percent of the research sample
		(N = 199)	
**Company size**	Very large (more than 5,000 employees)	41	21%
	Large (251–5,000 employees)	82	41%
	Medium-sized (51–249 employees)	34	17%
	Small (up to 50 employees)	42	21%
**Job level**	Line employee	120	60%
	Manager	79	40%
**Company ownership**	Local	88	44%
	Foreign	111	56%
**The region of origin of foreign investors**	Western Europe	111	100%
**Industry**	Construction	3	2%
	Education	7	4%
	Finance and insurance	8	4%
	Retail and wholesale sales	18	9%
	Other services	25	13%
	ICT	16	8%
	Manufacturing	102	51%
	Agriculture	2	1%
	Government and public administration	4	2%
	Arts, entertainment, and publishing	1	1%
	Business services (technical or legal)	11	6%
	Hotel and food service	2	1%

For this paper, the chi-squared test was used. The chi-squared test analyzes the relationship between two categorical variables. It examines the significance of differences in percentage structures by comparing the observed values (i.e., those obtained in the study) with the expected ones (i.e., those assumed by the test if there were no relationships between variables). It is a well-known and widely used statistical test. Its application in this study allows other scholars to easy replicate the research. In the case of significant values of χ^2^, Cramer’s V–as the most common measures of strength–should be calculated [69]. The latter has been done in this study, as well.

## Results and discussion

In the case of the first research question–concerning the importance of environmental issues for an enterprise–the result was statistically significant (χ^2^ = 24.276; *p* = <0.001). Table 3 shows the distribution of responses.

**Table 3:** The distribution of responses to the question about the importance of environmental issues for the organization.

			How important are environmental issues for your organization?			Total
			Slightly important or unimportant	Moderately important	Important or very important	
**Firm ownership**	**Local**	**Count**	21	39	27	87
		**%**	24.1%	44.8%	31.0%	100.0%
	**Foreign**	**Count**	6	36	69	111
		**%**	5.4%	32.4%	62.2%	100,0%
**Total**		**Count**	27	75	96	198
		**%**	13.6%	37.9%	48.5%	100.0%

In foreign companies, environmental issues are increasingly perceived as important or very important. This result is consistent with previous research conducted by Albornoz et al. [63] in Argentina and Henriques and Sadorsky [20] in Hungary, among others.

Before discussing the detailed results referring to the hypotheses, it is worth presenting which technical practices are used in the surveyed companies and how often green HRM activities are used. In the first case, the respondents indicated whether a given practice is applied, and in the latter (green HRM) how often it is used. The survey lists 20 practices of a technical nature (connected with environmental management, environmental infrastructure, and environmental rules). The total (percentage) results for the entire research sample are shown S1 Fig.

When it comes to technical practices promoting PEB among employees, the results of the research conducted in Poland in 2020 are consistent with those presented by Zibarras et al. [27] eight years earlier for companies in the UK. In turn, a study by Bombiak and Marciniuk-Kluska [22] showed that on the way to sustainable development, it is necessary to use mainly technical activities to promote environmentally-friendly attitudes in the performance of one’s professional duties (such as reducing paper use or sorting waste). As the above-mentioned results indicate, the surveyed companies primarily use such hard practices as a lights-out policy, energy-efficient light bulbs, recycling of waste materials, and central recycling bins. However, one technical practice that is rarely used is electronic record-keeping. In Poland, electronic documents are used for electronic invoices (e-invoices), for example. Companies are increasingly moving away from paper invoices in favor of digital ones. As a result, they not only reduce costs, but also improve the flow of information. Unfortunately, Polish legislation sometimes requires the original documentation (a hard copy with a personal signature) to be stored. For example, this applies to forms for opting out of employee capital plans (the government program of additional long-term savings in the optional part of the Polish pension system) [70]. This is certainly an obstacle on the way towards the complete digitization of documents.

S2 Table presents the percentage of companies using technical practices and the results of the chi-squared test. According to the chi-squared test, the differences between local and foreign-owned companies were statistically insignificant in the case of 10 (50%) practices only, including a “lights-out” policy (*p* = 0.46), double-sided printing (*p* = 0.48), encouraging employees to commute by bicycle (*p* = 0.09), purchasing green energy (*p* = 0.18), discouraging business travel (*p* = 0.13), and using e-documentation (*p* = 0.76). At this point, it is worth emphasizing that encouraging employees to commute by bicycle or discouraging business travel is not related to green HRM (e.g., internal communication), but to “hard” internal regulations and/or appropriate infrastructure.

Eltayeb and Zailani [61] reported different findings than those mentioned above. They indicated that the biggest differences occurred between local and foreign companies in the area of eco-design and green purchasing. In turn, the studies by Faith et al. [18] showed differences in energy efficiency between local and foreign companies.

The present study revealed a relationship between company ownership and the formulation of an environmental policy (*p*<0.01), in favor of foreign companies. This is in line with previous studies directly related to environmental policy [71] or more generally to environmental management systems [19, 63, 64], though it is in contradiction to the study by Garcia et al. [66].

As shown in S1 Fig, a “lights-out policy” was used in most of the companies surveyed, regardless of ownership (over 80% of foreign companies and over 90% of Polish businesses). However, it is worth emphasizing here that, in general, many practices are rarely used in these companies (i.e., by less than 30% of the organizations from each type). For example, e-documentation is used in only 23.6% of the surveyed companies–including 25% of foreign and 20% of Polish companies.

In the case of the remaining 10 (50%) technical practices, for which significant differences were observed between the groups of companies, it can be concluded that

- they are more often used by the vast majority of the companies surveyed and
- the foreign-owned companies are the leaders in the use of these practices.

For example, the recycling of waste materials (*χ^2^* = 11.26; *p* = 0.001) and an environmental policy (*χ^2^* = 32.30; *p*<0.001) were reported in more than 90% of the foreign-owned companies. On the whole, these results do not confirm the first hypothesis.

The survey questionnaire listed 17 “soft” organizational practices. The frequencies of the various practices according to the respondents are summarized S2 Fig. The most common answer regarding the majority of the “soft” practices was that they are never or rarely used.

As indicated in the theoretical part of the article, many authors emphasize the importance of training and remuneration in developing PEB (e.g., [21, 28, 55, 56, 72]. Masri and Jaaron [60] found that the most influential practice in the context of environmental organizational performance was not training, but recruitment and selection. Haddock-Millar et al. [32], on the other hand, pointed to managers’ influence in developing pro-environmental attitudes in employees. Meanwhile, “soft” practices are generally rarely used in the companies in question. This confirms that these organizations still have a lot of work ahead on the path towards becoming sustainable, i.e., operating on the principles of human and ecological sustainability, not only internally but also in a wider context [73].

As previously observed by Bombiak and Marciniuk-Kluska [22] and as the present study shows, green training practices are undervalued in Poland. This applies to both the low frequency of green training among the companies surveyed (59.8% of them never/rarely use it) and the assessment of its effectiveness in developing PEB. Green recruitment and selection are never/rarely used in 85.9% of the surveyed companies. In addition, the use of recruitment and selection criteria that take into account candidates’ attitudes towards environmental protection was assessed as the least effective practice in developing employees’ PEB, which is in line with the previous research done on young Polish companies by Bombiak and Marciniuk-Kluska [22].

The findings concerning the frequency of using green HRM practices broken down into Polish and foreign-owned companies are presented S3 Table. The statistical analysis revealed significant differences between local and foreign-owned companies in the frequency of the vast majority of them–as many as 15 out of 17 practices (88%)–which confirms the second hypothesis.

It is in the foreign-owned companies that the employees more often, for example, experience active championing by senior management (*χ^2^* = 17.28, *p*<0.01), participate in training courses aimed at developing environmental awareness (*χ^2^* = 17.18, *p*<0.01), or are covered by individual (*χ^2^* = 17.15, *p*<0.01) and team (*χ^2^* = 17.60, *p*<0.01) incentive programs. This is in line with results from a study conducted by Darnall et al. [71], who found that foreign-owned companies introduce pro-ecological “soft” practices more often (including those related to green training and employee remuneration).

The issue of environmental management practices implemented by companies with different ownership types is a complex one. The scope of the practices depends on the intentions of enterprises to take up eco-friendly actions. As presented in the Introduction, the stakeholder theory emphasizes the role of different groups who have some interest in the company’s actions. Foreign companies are likely to face a wider range of pressure from stakeholders both domestically and internationally, and this explains their commitment to environmental issues [71, 74].

Taking these findings into account, one can state that in 2002, Polish companies had fewer well-developed HRM programs than foreign-owned companies [75] and in 2020, there is still a clear difference between these two groups of companies–in the context of their pro-environmental practices.

The first implication for practice concerns the differences between Polish and foreign-owned companies. The research presented herein may prove to be important for domestic companies and foreign investors. The local companies (their managers)–based on the research findings–can take appropriate steps (following the example of foreign-owned companies) that can lead towards sustainable development. The article can inform foreign investors on the approach for developing PEB in Polish companies which already are or may become their business partners. This knowledge can be useful in the case of planned mergers or acquisitions.

Policymakers could also benefit from this study by finding justification to develop appropriate policies/interventions (i.e., that would enable the transfer of organizational practices) to make all firms, irrespective of their ownership, contribute equitably towards environmental sustainability.

Poland ranks 38th in the list of countries (and dependencies) by population (the current population is 37,833,050) [76]. Therefore, its impact on sustainable development–measured by the number of citizens–is meaningful. Poland has a developed market and the sixth-largest economy in the European Union and the tenth-largest in all of Europe (by nominal GPD). When Poland sets an example in safeguarding the planet, the rest of the under-developed and developing nations will follow.

Finally, countries willing to improve their environmental performance should analyze which countries mostly invest in Poland and encourage foreign investors from these countries to establish subsidiaries there.

The relative rarity of the practices in question in Polish-owned companies may result from a lack of knowledge about their potential and the proper ways to implement them. Public schools–as the main institutions responsible for the education of Polish society–should provide Polish entrepreneurs and managers with knowledge about the range of organizational practices which can be instituted to promote employees’ PEB.

## Conclusions and directions for further research

This study addresses emerging topics such as environmental sustainability and employees’ PEB. It extends the current state of knowledge by examining how much the firm’s ownership influences the degree to which organizational practices that promote PEB among employees are used.

The research shows that although in foreign companies environmental issues are increasingly perceived as important or very important, many of the practices which are considered in the literature as being of the utmost importance for shaping employees’ eco-friendly behaviors are rarely used in either local or foreign companies. However, it was empirically proven that the ownership significantly matters in the context of most (88%) “soft” practices that promote employees’ PEB, indicating that they are more often used by foreign-owned companies. In turn, only 50% of technical practices are significantly more often used in foreign-owned companies. This provides many practical implications, as presented in this study–for local companies, foreign investors, policymakers, and educational institutions–which will contribute to spreading these practices.

There are some limitations of this study which can nonetheless serve as the basis for further research. Firstly, the findings gathered from the snowball sampling method are not generalizable [77]. To overcome this limitation, further research should be based on a representative sample of the population.

Secondly, the article makes use of research conducted in Poland. Similar research could be carried out in other countries of the Visegrad Group to study whether their domestic companies also apply particular practices aimed at developing PEB less frequently than foreign-owned ones. It would also be scientifically interesting to compare whether a local business begins to implement more of these practices when starting a foreign expansion, perhaps due to pressure from the new stakeholders. Finally, as discussed in the article, the results of this research conducted in Poland in 2020 are consistent with those presented by Zibarras et al. [27] eight years earlier for companies in the UK. Therefore, it would be well worth comparing the situation in several countries with different levels of environmental protection spending, by conducting research that covers the same period.

Thirdly, the research sample included mainly large manufacturing companies. Wagner [10] found that company size matters when it comes to the propensity to introduce environmental training. Large companies institute educational programs more often. In turn, González-Benito and González-Benito [78] found a significant relationship between the industry and environmental proactivity. Guerci et al. [79] reported that manufacturing firms–perceived as greater polluters–tend to undertake more environmental activities and have better environmental performance than service companies. Therefore, within the framework of further research, it is worth considering the problem of correlations between other moderating/mediating characteristics of a company (the size and industry, as well as its position in the value chain, for example) and the application of practices aimed at developing PEB. Furthermore, the possible concentration of foreign investment in industries and companies of specific sizes can be used as a potential variable in further studies.

Fourthly, this article adopts a research instrument created by other authors to collect employees’ opinions. The issue of company ownership was added to the primary list of questions. Ownership can be difficult to recognize in practice [80] and it is possible that some respondents did not have sufficient knowledge to determine an accurate response. Therefore, other research methods including an analysis of the companies’ financial reports should be applied in further research.

## Supporting information

S1 Fig Technical practices which promote PEB in the surveyed companies. (TIF)

S2 Fig The frequency at which “soft” organizational practices are used in the surveyed companies. (TIF)

S1 Table Variables linked with company ownership in previous studies–based on Balasubramanian et al. (2021).
(DOCX)

S2 Table Results on the application of “technical” practices related to environmental management. (DOCX)

S3 Table Results on the frequency of green HRM practices in the surveyed organizations. (DOCX)
