# Supplementary figures and images for: Organizational practices promoting employees’ pro-environmental behaviors in a Visegrad Group country: How much does company ownership matter?
Source: PLoS One. 2022 Feb 3;17(2):e0261547. doi: 10.1371/journal.pone.0261547 (PMC8812892; doi:10.1371/journal.pone.0261547)

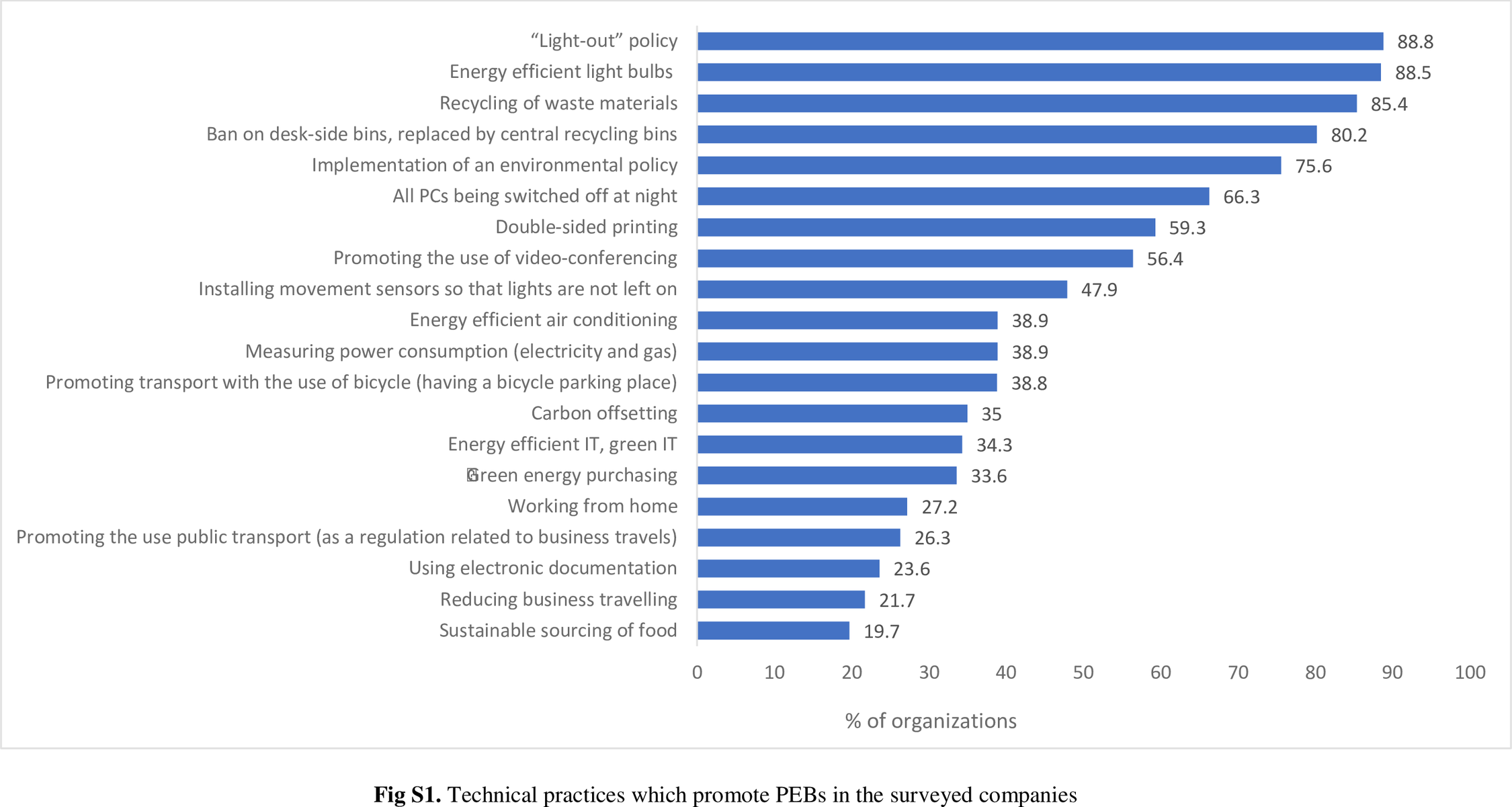

Supplement: S1 Fig — (TIF) [file pone.0261547.s001.tif]

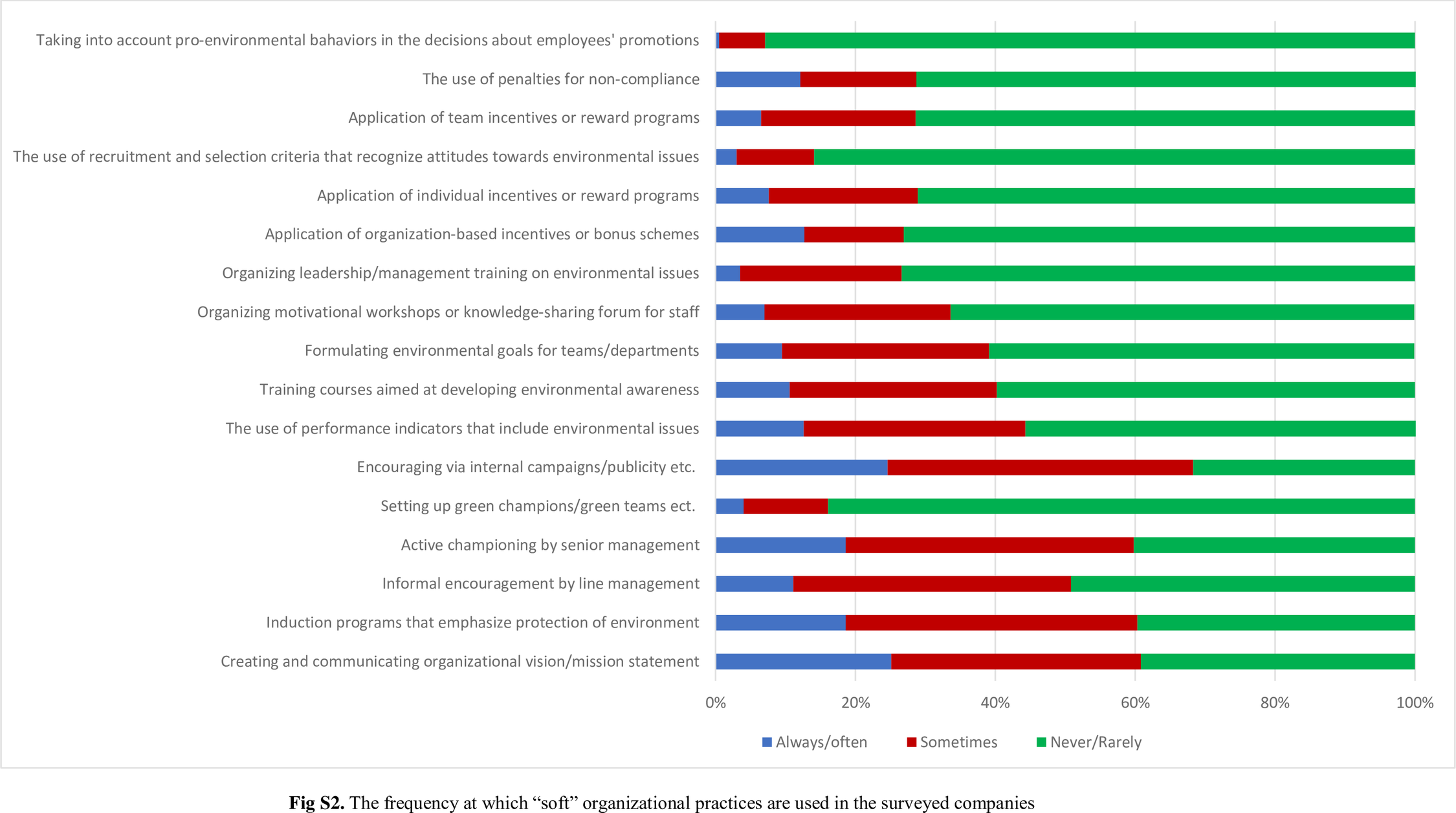

Supplement: S2 Fig — (TIF) [file pone.0261547.s002.tif]
