# Supplementary material for: Organizational practices promoting employees’ pro-environmental behaviors in a Visegrad Group country: How much does company ownership matter?
Source: PLoS One. 2022 Feb 3;17(2):e0261547. doi: 10.1371/journal.pone.0261547 (PMC8812892; doi:10.1371/journal.pone.0261547)
Supplement: S1 Table — (2021). (DOCX) [file pone.0261547.s003.docx]

**S1 Table.** Variables linked with company ownership in previous studies – based on (Balasubramanian et al., 2021)

|  | **Technical pro-environmental practices** | | | | | | | | | | | **"Soft" green practices** |
| --- | --- | --- | --- | --- | --- | --- | --- | --- | --- | --- | --- | --- |
| **Author** | **Eco-design** | **Green purchasing** | **Green manufacturing/ use of green technologies** | **Environmental  packaging** | **Environmental  transportation** | **Waste management** | **Product end-of-life  environmental practices** | **Environmental management systems and  ISO 14001** | **Environmental  disclosure/ reporting** | **Environmental  auditing** | **Environmental  related R&D** | **Green  training** |
| Christmann and Taylor (2001) |  |  |  |  |  |  |  | X |  |  |  |  |
| King and Shaver (2001) |  |  |  |  |  | X |  |  |  |  |  |  |
| Andonova (2003) |  |  | X |  |  |  |  | X |  | X |  |  |
| Cormier, Magnan, and  Van Velthoven (2005) |  |  |  |  |  |  |  |  | X |  |  |  |
| Henriques and Sadorsky  (2006) |  |  |  |  |  |  |  |  | X | X |  | X |
| Luken, Van Rompaey, and  Zigova (2008) |  |  | X |  |  |  |  |  |  |  |  |  |
| Albornoz, Cole, Elliott, and  Ercolani (2009) |  |  | X |  |  | X | X | X |  |  |  |  |
| Eltayeb and Zailani (2009) | X | X |  |  |  |  | X |  |  |  |  |  |
| Garcia, Bluffstone, and  Sterner (2009) |  |  |  |  |  |  |  | X |  |  |  |  |
| Darnall, Henriques, and  Sadorsky (2010) |  |  |  |  |  |  |  |  | X | X | X | X |
| Qi et al. (2011) |  |  |  |  |  |  |  | X |  |  |  |  |
| Abdo and Al-Drugi (2012) |  |  |  |  |  |  |  |  | X |  |  |  |
| Zhu, Cordeiro, and Sarkis (2012) |  |  |  |  |  |  |  | X |  | X |  |  |
| Tambunlertchai, Kontoleon,  and Khanna (2013) |  |  |  |  |  |  |  | X |  |  |  |  |
| Zhu and Geng (2013) | X | X | X | X | X |  | X |  |  |  |  |  |
| Li and Chan (2016) |  |  | X |  |  |  |  | X |  |  |  |  |
| Welbeck, Owusu, Bekoe, and Kusi  (2017) |  |  |  |  |  |  |  |  | X |  |  |  |
| Faith, Fagbenle, Amusan, and  Adedeji (2018) |  | X | X |  |  | X |  |  |  |  |  |  |
| Jensen and Berg (2012) |  |  |  |  |  |  |  |  | X |  |  |  |
| Yang et al. (2020) |  |  |  |  |  |  |  |  | X |  |  |  |
