# Supplementary material for: Organizational practices promoting employees’ pro-environmental behaviors in a Visegrad Group country: How much does company ownership matter?
Source: PLoS One. 2022 Feb 3;17(2):e0261547. doi: 10.1371/journal.pone.0261547 (PMC8812892; doi:10.1371/journal.pone.0261547)
Supplement: S2 Table — (DOCX) [file pone.0261547.s004.docx]

**S2 Table**. Results on the application of “technical” practices related to environmental management

| **No.** | **Organizational environmental (“technical”) practices** | **Origin of company capital** | | ***χ²* (df)** | ***p*** | ***Phi*** |
| --- | --- | --- | --- | --- | --- | --- |
|  |  | Local | Foreign |  |  |  |
|  |  | % | % |  |  |  |
| T1 | Recycling of waste materials | 74.7% | 93.4% | 11.26 | 0.001 | 0.26 |
| T2 | Formulating an environmental policy | 53.4% | 92.6% | 32.30 | <0.001 | 0.45 |
| T3 | Switching off all PCs at night | 76.9% | 58.0% | 6.20 | 0.013 | 0.20 |
| T4 | Introducing a “lights-out” policy | 91.4% | 86.8% | 0.56 | 0.456 | 0.07 |
| T5 | Double-sided printing | 56.0% | 62.2% | 0.50 | 0.478 | 0.06 |
| T6 | Using energy-efficient light bulbs | 82.3% | 95.0% | 3.70 | 0.054 | 0.20 |
| T7 | Installing motion sensors so that lights are not left on | 27.4% | 64.4% | 24.09 | <0.001 | 0.37 |
| T8 | Promoting the use public transport (“hard” rules in e.g. business travel policy) | 11.5% | 38.1% | 14.45 | <0.001 | 0.30 |
| T9 | Promoting transport with the use of bicycle (providing parking place for cyclists) | 31.2% | 44.9% | 2.91 | 0.088 | 0.14 |
| T10 | Measuring power consumption (electricity and gas) | 28.1% | 50.0% | 5.45 | 0.020 | 0.22 |
| T11 | Promoting the use of video-conferencing | 35.8% | 73.5% | 24.08 | <0.001 | 0.38 |
| T12 | Banning desk-side bins and replacing them with central recycling bins | 69.0% | 89.1% | 11.16 | 0.001 | 0.25 |
| T13 | Purchasing green energy | 27.5% | 39.7% | 1.76 | 0.184 | 0.13 |
| T14 | Promoting work from home | 17.0% | 35.5% | 7.41 | 0.006 | 0.21 |
| T15 | Reducing business travelling | 15.8% | 26.7% | 2.27 | 0.132 | 0.13 |
| T16 | Using energy efficient IT/Green IT | 14.3% | 60.5% | 21.00 | <0.001 | 0.48 |
| T17 | Sustainable sourcing of food | 12.9% | 25.6% | 3.11 | 0.078 | 0.16 |
| T18 | Using electronic documentation | 22.0% | 25.0% | 0.09 | 0.759 | 0.04 |
| T19 | Carbon offsetting, e.g., with potted plants provided by the employer | 31.6% | 37.9% | 0.47 | 0.494 | 0.07 |
| T20 | Using energy-efficient air conditioning systems | 33.3% | 47.4% | 1.34 | 0.246 | 0.14 |
|  | *χ²* – result of chi-squared test; *Phi* – Phi indicator | | | | | |
