# Supplementary material for: Organizational practices promoting employees’ pro-environmental behaviors in a Visegrad Group country: How much does company ownership matter?
Source: PLoS One. 2022 Feb 3;17(2):e0261547. doi: 10.1371/journal.pone.0261547 (PMC8812892; doi:10.1371/journal.pone.0261547)
Supplement: S3 Table — (DOCX) [file pone.0261547.s005.docx]

**S3 Table.** Results on the frequency with which green HRM practices are used in the surveyed organizations

| **No.** | **Organizational “soft” practices which promote pro-environmental behaviors** | **Origin of company capital (% calculated within the subgroup)** | | | | | | ***χ^2^*** | ***V*** |
| --- | --- | --- | --- | --- | --- | --- | --- | --- | --- |
|  |  | **Polish** | | | **Foreign** | | |  |  |
|  |  | **Never** | **Rarely** | **Often** | **Never** | **Rarely** | **Often** |  |  |
| S1 | Creating and communicating the organization’s vision/mission statement | 55.7% | 27.3% | 17.0% | 26.1% | 42.3% | 31.5% | 18.16** | 0.30 |
| S2 | Using induction programs that emphasize protection of the environment | 60.2% | 30.7% | 9.1% | 23.4% | 50.5% | 26.1% | 29.01** | 0.38 |
| S3 | Managers informally encouraging employees | 58.0% | 33.0% | 9.1% | 42.3% | 45.0% | 12.6% | 4.79 | 0.16 |
| S4 | Active championing by senior management | 55.7% | 34.1% | 10.2% | 27.9% | 46.8% | 25.2% | 17.28** | 0.30 |
| S5 | Setting up green champions/green teams, etc. | 95.5% | 4.5% | 0.0% | 74.8% | 18.0% | 7.2% | 16.23** | 0.29 |
| S6 | Encouraging through internal campaigns/publicity, etc. | 46.6% | 43.2% | 10.2% | 19.8% | 44.1% | 36.0% | 24.40** | 0.35 |
| S7 | Using performance indicators that include environmental issues | 77.3% | 15.9% | 6.8% | 38.7% | 44.1% | 17.1% | 29.57** | 0.39 |
| S8 | Using training courses aimed at developing environmental awareness | 75.0% | 21.6% | 3.4% | 47.7% | 36.0% | 16.2% | 17.18** | 0.29 |
| S9 | Formulating environmental goals for teams/departments | 79.5% | 17.0% | 3.4% | 45.9% | 39.6% | 14.4% | 23.79** | 0.35 |
| S10 | Organizing motivational workshops or a knowledge-sharing forum for staff | 77.3% | 17.0% | 5.7% | 57.7% | 34.2% | 8.1% | 8.70* | 0.21 |
| S11 | Using leadership/management training on environmental issues | 84.1% | 14.8% | 1.1% | 64.9% | 29.7% | 5.4% | 9.77** | 0.22 |
| S12 | Using organization-based incentives or bonus schemes | 82.6% | 11.6% | 5.8% | 65.8% | 16.2% | 18.0% | 8.27** | 0.21 |
| S13 | Using individual incentives or reward programs | 86.0% | 11.6% | 2.3% | 59.5% | 28.8% | 11.7% | 17.15** | 0.30 |
| S14 | Using recruitment and selection criteria that recognize attitudes towards environmental issues | 92.0% | 4.5% | 3.4% | 81.1% | 16.2% | 2.7% | 6.82** | 0.19 |
| S15 | Applying team incentives or reward programs | 86.4% | 11.4% | 2.3% | 59.5% | 30.6% | 9.9% | 17.60** | 0.30 |
| S16 | Using penalties for non-compliance | 85.2% | 10.2% | 4.5% | 60.4% | 21.6% | 18.0% | 15.48** | 0.28 |
| S17 | Taking into account pro-environmental behavior in decisions about employee promotions | 96.5% | 3.5% | 0.0% | 90.1% | 9.0% | 0.9% | 3.23 | 0.13 |

*χ²* - result of chi-squared test; *V* - V Cramer's indicator; *, p < 0,05,**, p < 0,01
